# Anticipatory grief among caregivers of people living with dementia: A scoping review

**DOI:** 10.1017/S1478951526102478

**Published:** 2026-05-06

**Authors:** Nury Alejandra Rodriguez Colmenares, Loreli Alvarez, James Gilbreath, Adelais Markaki, Julie Schexnayder, Frank Puga

**Affiliations:** 1 School of Nursing, University of Alabama at Birmingham, Birmingham, AL, USA; 2 UAB Libraries, University of Alabama at Birmingham, Birmingham, AL, USA

**Keywords:** Anticipatory grief, pre-death grief, dementia grief, dementia caregivers, mental health

## Abstract

**Objectives:**

As Alzheimer’s disease and related dementias (ADRD) progress, family caregivers may experience grief before the death of the person living with ADRD. This type of grief is commonly referred to as anticipatory grief, which can contribute to increased psychological distress (i.e., depressive and anxiety symptoms) and potentially affect caregivers’ long-term mental health. This scoping review aimed to synthesize evidence on risk and resilience factors associated with anticipatory grief, its relationship with caregiver mental health, and psychosocial interventions targeting anticipatory grief among caregivers of people living with ADRD.

**Methods:**

Guided by the Stress Process Model and the Grief-Stress Model, a literature search was conducted in PubMed, CINAHL, Embase, Scopus, Web of Science, and PsycINFO in May 2025.

**Results:**

Thirty articles met the inclusion criteria. Caregiver characteristics, such as being a spousal caregiver and greater caregiving involvement, were associated with higher levels of anticipatory grief. Caregiving-related stressors and relationship changes across the ADRD trajectory were consistently linked to anticipatory grief across studies, while psychosocial resources, such as adaptive coping and social support, were generally associated with lower levels of anticipatory grief. Anticipatory grief was consistently associated with depressive symptoms. Intervention studies were limited, but those focused on acceptance and preparedness showed potential for reducing anticipatory grief.

**Significance of results:**

Anticipatory grief represents an important dimension of caregiver mental health that reflects ongoing loss. Conceptualizing anticipatory grief within caregiving stress frameworks highlights how vulnerability to distress may emerge from the interplay between caregiving stressors, relationship changes, and psychosocial resources. This conceptual framing may inform future research and palliative care interventions to support the well-being of family caregivers across the dementia trajectory.

## Introduction

Family caregivers of older adults living with Alzheimer’s disease and related dementias (ADRD) are uniquely at risk for sustained psychological distress (i.e., depressive and anxiety symptoms) given the progressive nature of the disease and increasing caregiving demands over time (Fonareva and Oken 2014; Cho et al. 2024). Grief experienced while the person living with ADRD is still alive may contribute to increased psychological distress and adversely affect the long-term mental health of family caregivers (Rando 1986; Lindauer and Harvath 2014; Dehpour and Koffman 2023; Manevich 2025). This form of grief is commonly referred to as anticipatory grief, although related terms such as pre-death grief and dementia-related grief are also used in the literature. Unlike bereavement following death, anticipatory grief reflects caregivers’ emotional responses to cumulative and ongoing losses related to progressive cognitive decline and changes in the caregiver–care recipient relationship, with potential implications for caregiver well-being and the quality of care provided to the person living with ADRD (Rando 1986).

Anticipatory grief is highly prevalent among caregivers of people living with ADRD, with rates ranging from 10% to 32% (Crawley et al. 2023). The elevated prevalence likely reflects the progressive and prolonged nature of dementia, which exposes caregivers to gradual losses in communication and relationship quality, stress related to managing behavioral and psychological symptoms of dementia (BPSD), and increasing caregiving burden. Prior research has identified different caregiver and contextual factors associated with higher levels of anticipatory grief, including caregivers’ sex and their relationship to the person living with ADRD, caregiving intensity (i.e., time spent providing care and number of caregiving-related tasks), dementia severity, and available social support (Chan et al. 2013; Crawley et al. 2023; Wangliu and Che 2025). However, prior studies have typically examined these factors in isolation and have used heterogeneous definitions and measures of anticipatory grief, thereby limiting understanding of how caregiving-related stressors, relationship changes, and psychosocial resources are jointly associated with anticipatory grief and caregiver mental health outcomes.

Prior reviews have addressed specific aspects of anticipatory grief in ADRD caregiving, such as the social environment, measurement of pre-death grief, or the prevalence and correlates of grief before and after death (Nielsen et al. 2016; Crawley et al. 2023; Dehpour and Koffman 2023; Ng et al. 2025). Additionally, prior reviews of psychosocial interventions for caregiver grief have not primarily focused on family caregivers of people living with ADRD and have largely focused on caregivers of individuals residing in long-term care settings (Wilson et al. 2017). Although these reviews have advanced understanding of anticipatory grief, the literature in this area has grown rapidly in recent years. In addition, prior reviews have largely examined individual domains in isolation. The present review synthesizes evidence across caregiving-related stressors, relationship changes, and psychosocial resources within caregiving stress frameworks to better understand mechanisms associated with anticipatory grief and caregiver mental health outcomes.

### Theoretical framework

The Stress Process Model provides a useful framework for understanding how caregiving-specific risk and resilience factors influence caregivers’ psychological outcomes, including anticipatory grief (Pearlin et al. 1990). The model posits that relationships among caregivers’ background and context, caregiving primary and secondary stressors, and mediating factors contribute to psychological and physical health outcomes. Background and context include caregiver sociodemographic characteristics, such as age and gender. Primary stressors refer to demands directly related to caregiving, such as ADRD severity, BPSD management, and functional limitations, whereas secondary stressors are those indirectly related to the caregiving role, such as financial strain and competing family or work demands. Finally, mediating factors, such as coping strategies and social support, represent resources that can mitigate the adverse effects of caregiving-related stressors on caregivers’ health.

Building on the Stress Process Model, the Grief-Stress Model of caregiving conceptualizes anticipatory grief as a central outcome of caregiving stress, driven primarily by ambiguous loss and role overload (Noyes et al. 2010). Ambiguous loss refers to changes in the caregiver–care recipient relationship (e.g., changes in communication or companionship over time), while role overload reflects caregiver losses associated with the demands of caregiving (e.g., loss of personal freedom, personal identity, and social connections). This model emphasizes that caregiver grief is influenced by the perceived significance of these losses and available coping resources.

**Figure 1.**
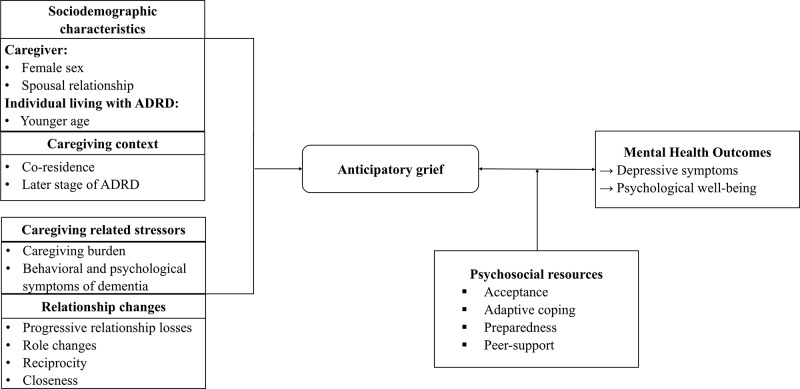
Sociodemographic, caregiving, and psychosocial factors associated with anticipatory grief.

Guided by the Stress Process Model and the Grief-Stress Model of Caregiving, the purpose of this scoping review was to synthesize evidence on risk and resilience factors associated with anticipatory grief, its relationship with caregiver mental health, and psychosocial interventions targeting anticipatory grief among family caregivers of people living with ADRD. Given the breadth of the topic, the conceptual heterogeneity of anticipatory grief, and the wide range of study designs and outcomes in recent literature, a scoping review was appropriate to comprehensively map what is known about anticipatory grief in dementia caregiving. This review extends prior research by organizing existing evidence within caregiving stress frameworks to identify potentially modifiable risk and resilience factors associated with anticipatory grief and caregiver mental health outcomes (Figure 1). The following research questions guided this scoping review:

1. What caregiving-related stressors, relationship changes, and psychosocial resources are associated with anticipatory grief among family caregivers of people living with ADRD?
2. How is anticipatory grief associated with caregiver mental health outcomes among family caregivers of people living with ADRD?
3. What psychosocial interventions have been evaluated to address anticipatory grief and caregiver mental health outcomes among family caregivers of people living with ADRD?

## Methods

This scoping review was conducted following the Arksey and O’Malley (2005) methodological framework, which includes 5 key stages: specifying the research question; identifying relevant literature; selecting studies; mapping the data; and summarizing, synthesizing, and reporting the results. Reporting of the review adhered to the Preferred Reporting Items for Systematic Reviews and Meta-Analyses extension for Scoping Reviews (Tricco et al. 2018).

### Search strategy

An academic reference librarian (JG) conducted a comprehensive search across 6 electronic databases: PubMed, CINAHL, Embase, Scopus, Web of Science, and PsycINFO. To extend and update prior reviews that included earlier literature, the search was limited to publications from January 2014 to May 2025 and was completed on May 23, 2025. This timeframe was selected to capture recent studies published following earlier reviews and reflect more recent conceptual and empirical developments in anticipatory grief research in dementia caregiving. The search terms included, but were not limited to, “caregivers,” “dementia,” “Alzheimer’s disease,” “pre-death grief,” and “anticipatory grief.” Detailed search terms for each database are available in Supplementary Material 1.

### Eligibility criteria

To be included in the review, studies needed to (i) examine anticipatory grief among family caregivers of people living with ADRD and (ii) report empirical findings related to caregiving-related stressors, relationship changes, psychosocial resources, caregiver mental health outcomes, or psychosocial interventions targeting anticipatory grief. Peer-reviewed original research articles written in English, with full-text access, were included. Studies focused exclusively on bereaved caregivers or post-bereavement outcomes were excluded.

### Selection of sources of evidence

For the screening and selection process, search results were exported into Covidence, a web-based collaboration software designed to support literature reviews (Covidence 2025). Duplicate articles were removed by Covidence and manually by 2 independent reviewers (NARC and LAD). Both reviewers independently screened potential abstracts and full-text articles using the predefined inclusion criteria. A third reviewer (FP) resolved conflicts, as needed.

### Data extraction

Data from eligible studies were extracted in Covidence using a data extraction matrix that was developed a priori. Two reviewers (NARC and LAD) independently extracted study data, and then collaboratively refined and finalized the entries using an iterative process. Extracted data included study characteristics (e.g., authors, country, study design and aims, and anticipatory grief assessment), sample characteristics (e.g., sample size, age, sex, caregiver relationship to the care recipient, and race/ethnicity), and main findings (e.g., risk and protective factors associated with anticipatory grief). Additional details are presented in Table 1.

**Table 1.** Sampled study characteristics (*n* = 30)

Author, year (country)	Design	Sample CG: *n* Age: mean ± SD; range Sex: *n* (%) Relationship to the CR: *n* (%) Race/ethnicity: *n* (%)	Aim	AG assessment	Key findings
Argueta et al. (2024) (United States)	Cross-sectional	CG: 103 Age: 71.27 ± 8.22; N/A Sex: Female: 78 (76) Male: N/A Relationship to the CR: Spouses: 103 (100) Race/ethnicity: N/A	To understand how specific psychosocial caregiving experiences (caregiver burden and AG) contributed to depressive symptoms and how experiences of childhood trauma might moderate these relationships	Anticipatory Grief Scale (AGS)	AG was significantly associated with depressive symptoms The main effects of AG on depressive symptoms were strong for caregivers reporting high levels of childhood trauma
Brice et al. (2025) (United States)	Cross-sectional	CG: 111 Age: 71.22 ± 8.22; N/A Sex: Female: 85 (76.58) Male: 26 (23.42) Relationship to the CR: Spouses: 111 (100) Race/ethnicity: White: 91 (81.98) Asian: 4 (3.60) Black/African American: 13 (11.71) Other: 3 (2.70) Hispanic: 6 (5.41)	To examine the associations between loneliness, proinflammatory cytokine production, and depressive symptoms in ADRD spousal caregivers To test whether childhood trauma, anticipatory grief, or sleep quality would moderate these hypothesized relationships	AGS	Greater loneliness was significantly associated with more depressive symptoms among caregivers reporting high levels of AG and mean levels of AG, but not among caregivers reporting low levels of AG. AG moderated the relationship between loneliness and proinflammatory cytokine production and the relationship between loneliness and depressive symptoms
Champlin, B. (2020) (United States)	Qualitative, phenomenological, one-on-one interviews	CG: 12 Age: 71.27 ± 8.22; N/A Sex: N/A Relationship to the CR: Spouses: 10 Adult children: 2 Race/ethnicity: N/A	To describe the lived experience of informal caregivers (meaning and impact) who accompany loved ones as the loved ones receive a diagnosis of dementia	Participants were asked to return to the moment of receiving the diagnosis and describe it in detail	Caregivers felt grief before and at the time of the diagnosis, related to the loss of the person they had known and the realization that there would be more losses Caregivers perceived losses long before a formal diagnosis Some caregivers, when they received a formal diagnosis, felt that their observations were validated
Chaudhry et al. (2024) (Singapore)	Prospective cohort study	CG: 169 Age: 56.4 ± 9.7; 63–98 Sex: Female: 118 (69.8%) Male: N/A Relationship to the CR: Spouses: 20 (11.8) Adult children: 145 (85.5) Other: 4 (2.4) Race/ethnicity: N/A	To investigate the temporal association of caregiving stressors and caregiver AG, and its mediation by positive and negative caregiving experiences in severe dementia	Singapore version of the MM-CGI-SF	BPSD had a significant direct effect on AG Negative caregiving experiences mediated the effect of BPSD and co-residence on AG Emotional closeness did not have a significant direct or indirect effect on AG
Cheung et al. (2018) (China)	Cross-sectional	CG: 108 Age: 62.9 ± 12.6; N/A Sex: Female: 85 (78.7) Male: 23 (21.3) Relationship to the CR: Spouses: 54 (50) Adult children: 54 (50) Race/ethnicity: N/A	To compare AG levels between spousal and adult children’s caregivers of people at earlier or later stages of dementia To explore the relationship between AG level and subjective caregiving burden and well-being	Cantonese version of the MM-CGI-SF	Spousal caregivers experienced higher levels of AG Spousal caregivers who were caring for a person with later ADRD stages had higher levels of AG AG was negatively associated with well-being and positively associated with subjective caregiver burden
Duplantier et al. (2023) (United States)	Qualitative, semi-structured interviews	CG: 8 Age: N/A; 32–83 Sex: Female: 7 Male: 1 Relationship to the CR: Spouses: 4 Adult children: 4 Race/ethnicity: N/A	To identify barriers and facilitators to caregiver health and well-being, and potential strategies to facilitate better caregiver self-care	Caregivers were asked about their ability to manage their health and well-being	Caregivers appeared to experience AG as the person they loved changed, becoming psychologically absent while remaining physically present Profound changes in the caregiver-person living with ADRD relationship In older caregivers, religion and spirituality were connected closely with a divine power and represented a connection to the community
Gilsenan et al. (2023) (United Kingdom)	Cross-sectional	CG: 530 Age: 54 ± 12.9; N/A Sex: Female: 459 (86.6%) Male: N/A Relationship to the CR: Spouses: 125 Adult children: 167 Other: 137 Race/ethnicity: White/White British: 511(96.4) Other: 19 (3.6)	To explore which variables contribute to explaining the variation in the overall subjective burden experienced by caregivers	The MM-CGI-SF	Higher levels of AG were related to higher levels of caregiving burden
Han et al. (2025) (United States)	Randomized-controlled trial (RCT)	CG: 33 Age: 55.2 ± 12.7; 23–78 Sex: Female: 29 (88) Male: 4 (12) Relationship to the CR: Spouses: 8 (24) Other: 25 (76) Race/ethnicity: White: 24 (73) Black/African American or Hispanic: 9 (27)	To assess the effects of a videoconference-delivered, therapist-guided ACT program on reducing depressive symptoms and improving other mental health outcomes among family caregivers with depression	The Marwit–Meuser Caregiver Grief Inventory Brief Form (MM-CGI-BF)	No significant between-group differences were observed for other outcome measures in the posttest, and none were observed for any outcome measure in the 3-month follow-up For within-group comparisons, the ACT group reported significant improvements in AG in both posttest and 3-month follow-up
Kobiske et al. (2019) (United States)	Cross-sectional	CG: 104 (spousal) Age: 58.27 ± 11.2; 27–80 Sex: Female: 67 (65.26) Male: N/A Relationship to the CR: Spouses: 104 (110) Race/ethnicity: White: 98 (94.74) Asian: 2 (2.11) Black/African American: 1 (1.05) Hispanic: 2 (2.11)	To investigate the moderating effects of both personal resourcefulness and social resourcefulness on the relationship between AG and caregiver stress of caregivers of people diagnosed with YOD using the theoretical framework of Resilience Theory	The MM-CGI-SF	AG was associated with caregiver perceived stress The relation between AG and caregiver stress was only moderated by social resourcefulness. As social resourcefulness increased, the relationship between AG and caregiver perceived stress increased
Kotwal et al. (2024) (United States)	Secondary analysis of qualitative semi-structured interviews	Dementia Palliative Care study: Current CG: 16 Age: 65.8; 40–87 Sex: Female: 12 (75) Male: 4 (25) Relationship to the CR: Spouses: 3 Other: 13 Race/ethnicity: White: 9 (56) Asian: 3 (19) Black/African American: 2 (13) Other: 2 (13) Music and Dementia Caregiving study: Current CG: 17 Age: 68.3; 47–83 Sex: Female 12 (71) Male: N/A Relationship to the CR: N/A Race/ethnicity: White: 7 (41) Black: 4 (24) Asian: 4 (24) Other: 2 (11)	To understand how people with ADRD and caregivers perceive the impact of social isolation and loneliness	Losses within the dyadic relationship Caregiver adaptation to the disease and caregiving role	The extended, slow-motion AG process was emotionally taxing for caregivers and led to social withdrawal Caregivers described disruptions of meaningful dyadic relationships due to progressive cognitive and functional deficits, leading to AG Caregivers described developing or adapting shared social activities to preserve their relationship quality at various stages of the disease trajectory
Liew et al. (2020) (Singapore)	Prospective cohort study	CG: 178 56.6 ± 9.9; 27–80 Sex: Female: 130 Male: N/A Relationship to the CR: Spouses: 35 Adult children: 143 Race/ethnicity: Chinese: 152 Malay/Indian/Eurasian/Other: 26	To investigate the longitudinal effects of caregiver grief on caregiver depression, quality of life, and perceived positive aspects of caregiving To examine the potential effect modification of social service utilization	The MM-CGI	AG was associated with higher levels of depressive symptoms, poorer quality of life, and fewer positive aspects of caregiving Dementia care services did not reduce AG Participation in caregiver programs appeared to worsen AG Paid caregivers partially alleviated AG
Liew et al. (2019) (Singapore)	Cohort study	CG: 183 Sex: Female: 123 (67.2) Male: N/A Age: 53.1 ± 11; N/A Relationship to the CR: Spouses: 31 (16.9) Adult children: 152 (83.1) Race/ethnicity: Chinese: 158 (86.3) Malay: 15 (8.2) Indian/Eurasian/Others: 10 (5.5)	To compare the effects of baseline grief and burden on caregiver depression at baseline and 2.5 years later	The MM-CGI	AG had an independent effect on caregiver depression both at baseline and at 2.5 years Higher levels of AG amplified the effect of burden on caregiver depression at baseline Caregivers with lower levels of AG developed depression at a higher burden, while caregivers with high AG developed depression at a lower burden AG has a direct effect on depression over time, which is distinct from and not related to baseline depression
Liew et al. (2018) (Singapore)	Cross-sectional	CG: 394 Age: 53 ± 10.7 Sex: Female: 236 (59.9) Male: N/A Relationship to the CR: Spouses: 54 (13.7) Adult children: 340 (86.3) Race/ethnicity: Chinese: 341(86.6) Malay: 25(6.3) Indian/Euroasian/others: 28 (7.1)	To contrast the risk factors of AG and caregiver burden	The MM-CGI	Unique risks AG: younger age of the person living with ADRD, lower educational attainment of caregivers, and spousal caregivers Risk factors shared by AG and caregiver burden: later stage of dementia, BPSD, and primary caregiving role The stage of dementia had a stronger association with AG
Malhotra et al. (2024) (Singapore)	Prospective cohort study	CG: 215 Age: 56.7 ± 10.1 Sex: Female 149 (69.3) Relationship to the CR: Adult children: 179 (83.3) Race/ethnicity: N/A	To assess the bidirectional association of caregiver burden and AG with acute health care use (inpatient or emergency admission) among older adults with severe dementia	Singapore version of the MM-CGI-SF	Caregivers’ family support did not moderate the association between caregiving burden and anticipatory grief associations
Meichsner et al. (2018) (Germany)	RCT	Intervention CG: 19 Age: 63 ± 9.40; 45–82 Sex: Female: 14 (73.7) Male: N/A Relationship to the CR: Spouses: 15 (78.9) Adult children: 4 (21.1) Race/ethnicity: N/A Control CG: 18 Age: 61.17 ± 10.14; 14–76 Sex: Female: 15 (83.3) Male: N/A Relationship to the CR: Spouses: 11 (61.1) Adult children: 7 (38.9) Race/ethnicity: N/A	To evaluate the efficacy of an internet-delivered cognitive behavioral intervention for caregivers of people with dementia and examine acceptance of program characteristics	Caregiver Grief Scale (CGS)	Caregivers improved their ability to cope with AG and increased their use of emotional and social support resources No significant difference between the intervention group and control group at 8 weeks after baseline, or 5 months after baseline The structured writing assignment and the therapist’s guidance and validation have possibly enabled caregivers to recognize (anticipated) losses and acknowledge associated emotional pain
Meichsner et al. (2016) (Germany)	RCT	Intervention CG: 139 Control CG: 134 Age: 64.20 ± 11.04, 23–91 Sex: Female: 220 (80.6) Male: N/A Relationship to the CR: Spouses: 165 (60.4) Adult children: 104 (38.1) Other: 4 (1.5) Race/ethnicity: N/A	To examine whether a cognitive-behavioral intervention, including a grief intervention module, could increase caregiver coping with AG and whether these effects could be maintained as of a 6-month follow-up assessment	CGS	Caregivers in the intervention group reported lower levels of AG post-intervention Caregivers who continued providing care at home post-intervention had lower levels of AG Being a spousal caregiver was associated with higher levels of AG
Ménard et al. (2025) (Canada)	Multi-method qualitative study, semi-structured interviews	CG: 15 Age: 63.5 ± N/A; 36–83 Sex: Female: 12 (80) Male: 3 (20) Relationship to the CR: Spouses: 6 (40) Adult children: 7 (46.7) Other: 2 (13.7) Race/ethnicity: White: 13 South Asian: 1 Mixed race: 1	To illuminate the nuanced ways in which care partners engage in future planning	Experiences of care partners providing daily care to a person living with dementia	AG underscores the emotional challenges faced by care partners as they navigate the complexities of future planning, particularly in the context of changes in the person living with ADRD and the inevitability of their death Caregivers who felt more uncertainty around the ADRD trajectory, future planning, and end-of-life decisions felt overwhelmed For some caregivers, AG drove the urgency to make funeral arrangements and decisions about end-of-life care, as they felt compelled to prepare for their relative’s passing
Moore et al. (2023) (UK)	Mixed methods observational study using structured interviews	Quantitative-CG: 150 Age: 63 ± 12.1; N/A Sex: Female: 116 (77.3) Male: N/A Relationship to the CR: Spouses: 70 (46.6) Adult children: 72 (48.0) Other: 8 (5.3) Race/ethnicity: White British (87) Qualitative-CG: 16 Sex: Female: 9 Male: 7 Race/ethnicity: N/A Relationship to the CR: Spouses: 5 Adult children: 8 Other: 3	To identify strategies that help carers manage AG To examine the association between coping styles and AG intensity To describe the strategies and resources that carers identify using to help manage AG	The MM-CGI-SF Qualitative questions focused on grief, coping, and social support	Higher levels of AG were moderately associated with greater use of dysfunctional coping strategies Strategies to manage AG included embracing carer identity, using psychological strategies (e.g., acceptance and humor), and seeking formal or informal support
Moore et al. (2020) (UK)	Cross-sectional	CG: 150 Age: 63.0 ± 12.1; N/A Sex: Female: 116 (77.3) Male: 34 (22.7) Relationship to the CR: Spouses: 70 (46.7) Adult children: 72 (48) Other: 8 (5.3) Race/ethnicity: N/A	To examine the relationship between AG in caregivers of people with dementia and how well caregivers are prepared for death To explore other demographic factors associated with AG and whether there were different associated factors for the 3 subscales of the MM-CGI-SF	The MM-CGI-SF	Reduced social support and dementia knowledge increased levels of AG Females had higher levels of AG Caregivers, who reported greater declines in the closeness of their relationship with the person living with ADRD experienced higher levels of AG
Park and Galvin (2021) (United States)	Cross-sectional	LBD CG: 488 Age: 60.1 ± 10.9; N/A Sex: Female: 431 (88.7) Male: (11.3) Relationship to the CR: Spouses: 275 (56.3) Adult children: 166 (33.7) Race/ethnicity: White: 467 (96.7) Other: (3.3) AD CG: 81 Age: 59.1 ± 10.6; N/A Sex: Female: (88.8) Male: (11.2) Relationship to the CR: N/A Race/ethnicity: White: (91.1) Other: (8.9) Other CG: 145 Age: 60.9 ± 10.6 Sex: Female:(91.0) Male: (9.0) Relationship to the CR: N/A Race/ethnicity: White: (97.9) Other: (2.1)	To examine factors associated with AG in caregivers of older adults with LBD and compare their AG with that of caregivers of persons living with AD and other dementias	The Prolonged Grief-12	AG was associated with being the spouse of the care recipient, lower levels of caregiver well-being, lower psychological well-being, and higher levels of burden Caregiver perceived ability to provide care and be mindful of their own well-being was associated with AG No significant differences in levels of AG by type of dementia No significant differences in levels of AG scores by the stages of LBD
Paulson et al. (2024) (United States)	Pre- and post-intervention	CG: 76 Age: 40–64 (51.3) 65+ (47.4) Sex: Female: (81.6) Male: N/A Relationship to the CR: Spouses: 22 (28.9) Adult children: 40 (52.6) Other: 14 (18.3) Race/ethnicity: White: 55 (72.4) Asian: 2 (2.6) Black/African American: 11 (4.5) Hispanic: 6 (7.9) Other: characteristics 2 (2.6)	To examine caregiver preparedness as one mechanism by which the Savvy Caregiver Program confers benefits to caregivers	AGS	Greater preparedness was associated with lower levels of AG
Pérez-González et al. (2023) (Spain)	Cross-sectional	CG: 129 Age: 62.09 ± 10.89; 32–85 Sex: Female: 80 (67.8) Male: 38 (32.2) Relationship to the CR: Spouses: 39 (32.8) Adult children: 73 (61.3) Other: 7 (5.9) Race/ethnicity: N/A	To assess the relation between burden and AG, considering the possibility of social support and the risk of psychopathology	The MM-CGI	Higher levels of AG were associated with higher caregiving burden No significant direct effects of their relationship of social support on anticipatory grief The direct effect of AG on psychopathology was greater than its indirect effect on psychopathology through caregiver burden
Pérez-González et al. (2021) (Spain)	Cross-sectional	CG: 129 Age: 61.56 ± 10.78; 32–85 Sex: Female: 80 (67.8) Male: 38 (32.2) Relationship to the CR: Spouses: 39 (32.8) Adult children: 73 (61.3) Other: 7 (5.9) Race/ethnicity: N/A	To determine which characteristics of family caregivers are related to the presence of AG	The MM-CGI	Being female, spousal and the main caregiver were associated with higher levels of AG There was no relationship between the caregiver’s age and education level with AG Younger age of the individual living with ADRD, and later stage of ADRD were associated with increased AG Duration of dementia was not correlated with AG Caregiving duration (years) and time providing care (hours) were positively correlated with AG Caregiving burden was associated with higher levels of AG
Pote et al. (2018) (United States)	Cross-sectional	CG: 90 Age: 63 ± 10.89; 39–81 Sex: Female: 66 (67.8) Male: 24 (32.2) Relationship to the CR: Spouses: 90 (100) Race/ethnicity: White/Caucasian: (62.2) African American: (20) Asian American: (14.4) Hispanic/Latino: (1.1)	To determine the association of relational factors and spousal caregiver satisfaction with life and marriage and explore if AG moderated these associations	The MM-CGI-SF	Lower levels of avoidant attachment and anxious attachment, and higher levels of perceived closeness, were associated with lower levels of AG AG did not moderate the association of anxious attachment, avoidant attachment, and perceived closeness with marital and life satisfaction AG was negatively associated with marital satisfaction and life satisfaction
Risch et al. (2024) (Germany)	RCT	Total CG: 81 Age: 61.94 ± 11.17 Sex: Female: 45 (55.6) Male: N/A Relationship to the CR: Spouses: 43 (53.8) Race/ethnicity: N/A Intervention CG: 41 Age: 62.49 ± 12.28; N/A Sex: Female: 22 (53.7) Relationship to the CR: Spouses: 23 Other: 30 Race/ethnicity: N/A Control CG: 40 Age: 61.37 ± 10.04 Sex: Female: 23 (57.5) Relationship to the CR: Spouses: 20 Other: 28 Race/ethnicity: N/A	To investigate the effects of telephone-based ACT for family caregivers	CGS	Caregivers in the intervention group reported lower levels of AG and had fewer problems accepting their grief reaction at 6 months post-intervention Caregivers reported more utilization of coping resources and social support after 2 months, but not after 6 months post-intervention
Sánchez-Alcón et al. (2024) (Spain)	Cross-sectional	CG: 250 Age: 58.2 ± 12.7; 24–87 Sex: Female: 201 (80.4) Male: 49 (19.6) Relationship to the CR: Spouses: 79 (31.6) Adult children: 155 (62) Other: 16 (6.4) Race/ethnicity: N/A	To describe the relationship between depressive symptoms, caregiver strain, and social support with dementia grief in family caregivers of people with dementia	The MM-CGI-SF	Being female, lower education level, and later ADRD stage were associated with higher levels of AG High levels of depressive symptoms and caregiver strain, and low perceived social support were associated with higher levels of AG Depressive symptoms showed the most weight and influence on AG
Stevens-Neck et al. (2024) (United Kingdom)	Pre-post intervention	CG: 9 Age: 58 ± 10.26; N/A Sex: Female: 8 Relationship to the CR: Spouses: 7 Adult children: 2 Race/ethnicity: White British: 8 White Irish: 1	To help carers of people with rarer dementias, to explore and acknowledge grief and loss, and to process difficult emotions associated with these feelings	CGS	Caregivers described that acceptance of AG improved well-being Understanding the concept of AG helped caregivers with acceptance, as caregivers felt more entitled to grieve
Supiano et al. (2022) (United States)	Mixed-methods descriptive phenomenological study	Pre-loss CG: 54 Age: Range: *n* (%) 20–40: 28 (51.8) 41–60: 21 (38.9) 61–80: 5 (9.3) Sex: Female: 41 (76) Male: 12 (22) Relationship to the CR: Spouses: 1 (2) Adult children: 22 (44) Grandchild: 20 (40) Others: 7 (14) Race/ethnicity: White: 43 (79.6) Asian: 1 (1.9) Black/African American: 8 (14.8) Multiracial: 1 (1.9) Hispanic: 1 (1.9)	To evaluate the relationship between grief preparedness and grief experience	Inventory of Complicated Grief-revised Brief Grief Questionnaire	Caregivers reported higher AG when not prepared for the death of the person living with ADRD Those with few or poor-quality social support were less prepared and had poorer AG Caregivers’ perception that they were managing caregiving alone or with unsupportive others influenced caregivers’ AG experience
Türk et al. (2025) (Turkiye)	Cross-sectional	AD CG: 66 Age: 57.62 ± 11.46; N/A Sex: Female: 45 (68.2) Male: 21 (31.8) Relationship to the CR: Spouses: 23 (34.8) Adult children: 43 (65.2) Race/ethnicity: N/A HM CG: 66 Age: 51.42 ± 13.58; N/A Sex: Female: 40 (60.6) Male: 26 (39.4) Relationship to the CR: Spouses: 27 (40.9) Adult children: 39 (59.1) Race/ethnicity: N/A	To examine the prevalence and severity of AG in caregivers of patients with major neurocognitive disorder due to Alzheimer’s disease compared to hematological malignancies (HM), and to identify the characteristics associated with AG	The MM-CGI-SF	AD group results: AG was associated with caregiving burden, disease duration, and cognitive status AG did not differ with caregiver sex or education level Adult children presented higher scores on the W&FI subscale than spousal caregivers There was no statistically significant difference between the groups in terms of the severity of AG
Wangliu et al. (2024) (China)	Cross-sectional	CG: 320 Age: 54.7 ± 4.02; N/A Sex: Female 168 (52.5) Male: 152(47.5) Relationship to the CR: Spouses: 100 (41) Adult children: 112 (46) Other: 31 (13) Race/ethnicity: Han: (92.5) Other: (7.5)	To examine the relationship between caregiving burden and AG and to determine whether various coping strategies serve as mediators of this relationship	The Chinese version of the MM-CGI-SF	AG was associated with caregiving burden. Active coping mediated this association Active coping acted as a mediator between caregiving burden and AG Among females, active coping mediated the association between caregiving burden and AG

ACT = acceptance and commitment therapy, AD = Alzheimer’s disease, AG = anticipatory grief, BPSD = behavioral and psychological symptoms of dementia, CG = caregivers, LBD = Lewy Body Dementia, YOD = Young-onset dementia. The study by Pérez-González et al. (2023) was built upon the data collected in their earlier research from 2021 (Pérez-González et al. 2021). The study conducted by Moore et al. (2023) and Moore et al. (2020) used the same sample of caregivers. The study conducted by Liew et al. (2019) corresponds to the follow-up for the participants recruited in their previous study (Liew et al. 2018).

### Synthesis and reporting

A descriptive synthesis was conducted to summarize key findings across included studies. Studies were first characterized by design and sample characteristics. Findings were then organized and synthesized across the following key domains: (i) sociodemographic characteristics and caregiving context, (ii) caregiving-related stressors, (iii) relationship changes, (iv) psychosocial resources, (v) mental health outcomes, and (vi) intervention approaches. Quantitative results were synthesized narratively to summarize the direction and nature of associations across studies, while qualitative data were analyzed to identify recurrent themes and explanatory insights. Figure 1 presents the organizing framework for the results.

## Results

### Study selection and characteristics

**Figure 2.**
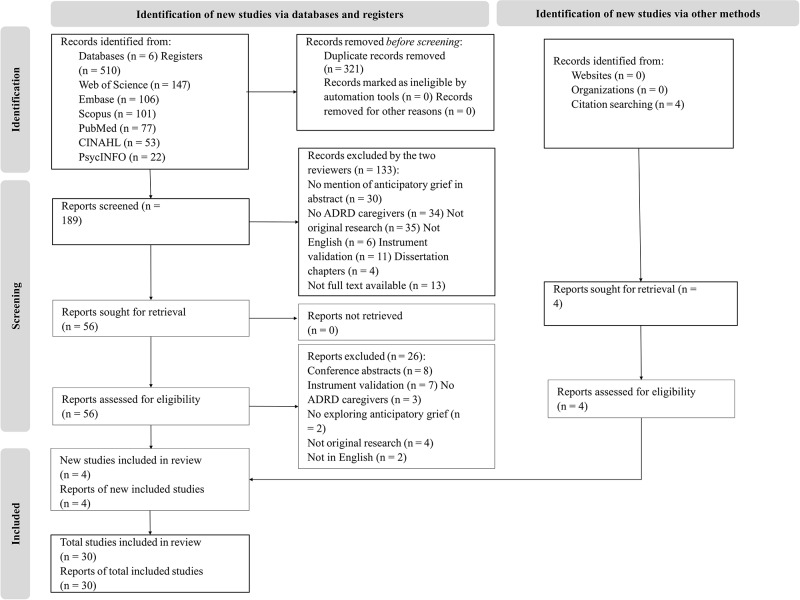
PRISMA flow diagram of study selection process.

The search resulted in 510 articles. After removing 321 duplicates, 189 articles were screened for eligibility. Of these, 133 were excluded after title and abstract screening. Thirty studies met the inclusion criteria and were included in the final review (Figure 2).

Of the included studies, 18 were quantitative, 4 were qualitative, 2 were mixed methods, and 6 were intervention studies. Almost half of the studies were cross-sectional (*n* = 14). Most of the studies were conducted in the United States (*n* = 11) and Singapore (*n* = 5). The most common anticipatory grief measures were the Marwit–Meuser Caregiver Grief Inventory Short Form (MM-CGI-SF) (*n* = 10) and the Marwit–Meuser Caregiver Grief Inventory (MM-CGI) (*n* = 7), including sub-scales measuring personal sacrifice and burden (PS&B), heartfelt sadness and longing (HS&L), and worry and felt isolation (W&FI).

Across all studies, 4,580 family caregivers of people living with ADRD were included. Sample sizes ranged from 103 to 530 in quantitative studies, 8 to 33 in qualitative studies, 106 to 150 in mixed methods studies, and 9 to 273 in intervention studies. The average age among caregivers ranged from 53 to 71 years. Caregivers were predominantly female (52.5–88.7%) and most commonly adult children (*n* = 2,106) or spouses (*n* = 1,669). Samples primarily included caregivers identifying as White or Asian. Additional study characteristics are presented in Table 1.

### Sociodemographic characteristics and caregiving context

Caregiver sociodemographic characteristics were frequently examined as variables associated with anticipatory grief. Two studies found no significant association between caregiver age and anticipatory grief (Park and Galvin 2021; Pérez-González et al. 2021). Findings regarding sex were mixed. Three studies reported that the caregiver’s sex was not associated with anticipatory grief (Meichsner et al. 2018; Park and Galvin 2021; Türk et al. 2025), while 3 studies reported that female caregivers had higher levels of anticipatory grief (Moore et al. 2020; Pérez-González et al. 2021; Sánchez-Alcón et al. 2024). Educational attainment showed similar mixed patterns. Two studies reported that lower caregiver educational attainment was associated with higher levels of anticipatory grief (Liew et al. 2018; Sánchez-Alcón et al. 2024), while other studies found no statistically significant differences by educational attainment (Pérez-González et al. 2021; Türk et al. 2025).

Relationship to the person living with ADRD demonstrated more consistent associations. Six studies found that spousal caregivers reported higher levels of anticipatory grief (Meichsner and Wilz 2016; Liew et al. 2018; Meichsner et al. 2018; Moore et al. 2020; Park and Galvin 2021; Pérez-González et al. 2021). However, one study reported that adult children had higher scores on the MM-CGI W&FI subscale compared to spousal caregivers (Türk et al. 2025). Caregivers who co-resided with the person living with ADRD reported more negative caregiving experiences, which were associated with higher levels of anticipatory grief (Chaudhry et al. 2024). Finally, greater caregiver involvement, including identifying as the primary caregiver, longer caregiving duration (i.e., number of years as a caregiver), and more daily hours spent providing care were associated with higher levels of anticipatory grief (Pérez-González et al. 2021).

Characteristics of the person living with ADRD were also associated with caregivers’ anticipatory grief. Three studies reported that the younger age of the person living with ADRD was associated with higher levels of anticipatory grief (Liew et al. 2018; Moore et al. 2020; Pérez-González et al. 2021). Six studies reported that dementia severity was associated with anticipatory grief (Cheung et al. 2018; Liew et al. 2018; Park and Galvin 2021; Pérez-González et al. 2021; Sánchez-Alcón et al. 2024; Türk et al. 2025). Further, caregivers of people with a later stage ADRD reported higher levels of anticipatory grief (Pérez-González et al. 2021; Sánchez-Alcón et al. 2024; Türk et al. 2025). Specifically, spousal caregivers of people in late-stage ADRD reported significantly higher PS&B and W&FI scores of the MM-CGI-SF than those caring for people in early stages and adult child caregivers of a person in either early or later ADRD stages (Cheung et al. 2018). However, among caregivers of people with Lewy Body Dementia, dementia stage was not associated with anticipatory grief (Park and Galvin 2021).

### Caregiving-related stressors and burden, relationship changes, and psychosocial resources associated with anticipatory grief

#### Caregiving-related stressors and burden

One study found that BPSD, established caregiving-related stressors, increased anticipatory grief directly and indirectly through negative caregiving experiences. Specifically, BPSD increased negative caregiving experiences, which in turn were associated with higher levels of anticipatory grief (Chaudhry et al. 2024). However, another study reported that BPSD were not significantly associated with anticipatory grief (Park and Galvin 2021). Nine studies reported that higher levels of anticipatory grief were associated with higher perceived caregiving burden (Cheung et al. 2018; Liew et al. 2018, 2020; Park and Galvin 2021; Gilsenan et al. 2023; Pérez-González et al. 2023; Sánchez-Alcón et al. 2024; Wangliu and Chen 2024; Türk et al. 2025). Among the 3 MM-CGI-SF subscales, the PS&B subscale had the strongest association with caregiving burden. After excluding the PS&B subscale, caregiving burden remained associated with HS&L and W&FI subscales (Gilsenan et al. 2023). Finally, Liew et al. (2018) found that caregiving burden and anticipatory grief were both associated with ADRD stage, BPSD, and caregiving role (i.e., being the primary caregiver).

#### Relationship changes

Three studies reported that ADRD family caregivers experienced anticipatory grief related to the loss of the person they had known and the realization of future losses (Champlin 2020; Duplantier and Williamson 2023; Moore et al. 2023). Caregivers described progressive losses in their relationship with the person living with dementia, including decreased reciprocity and emotional connection. Three studies described role changes in the relationship with the person living with ADRD (e.g., becoming the primary decision-maker, assuming responsibility for organizing their social life, or feeling like a separated spouse despite being married), which contributed to feelings of relationship loss (Duplantier and Williamson 2023; Moore et al. 2023; Kotwal et al. 2024). Spouses also described loss of reciprocity in the relationship, and daughters described becoming the “new parent” (Duplantier and Williamson 2023). As caregivers acknowledged relationship changes, they described adjusting their expectations of the relationship and adapting shared social activities to preserve relationship quality (Kotwal et al. 2024). Finally, anticipatory grief was negatively associated with marital satisfaction and life satisfaction (Pote and Wright 2018). However, the quality of the caregiver–care recipient relationship was not associated with caregiver anticipatory grief (Chaudhry et al. 2024).

#### Psychosocial resources

Across studies, psychosocial resources were consistently examined as potential protective factors associated with lower levels of anticipatory grief; however, findings varied by domain. Higher psychological well-being, quality of life, and resilience were associated with lower levels of anticipatory grief (Cheung et al. 2018; Liew et al. 2020; Park and Galvin 2021; Gilsenan et al. 2023). Similarly, greater perceived closeness was associated with lower levels of anticipatory grief (Pote and Wright 2018; Moore et al. 2020). Further, among spousal caregivers, low levels of avoidant (i.e., experiencing discomfort with intimacy and preferring independence) and anxious attachments (i.e., fearing rejection and abandonment) were associated with lower levels of anticipatory grief (Pote and Wright 2018).

Coping strategies emerged as a predominant psychosocial factor associated with anticipatory grief. Specifically, greater anticipatory grief was associated with higher use of dysfunctional coping, such as denial, avoidance, and self-blame (Kobiske et al. 2019; Duplantier and Williamson 2023; Moore et al. 2023; Kotwal et al. 2024; Wangliu and Chen 2024). Adaptive coping, such as acceptance, humor, carer identity, and seeking support, was found to help caregivers manage anticipatory grief (Moore et al. 2023). Active coping mediated the association between caregiving burden and anticipatory grief among female ADRD caregivers (Wangliu and Chen 2024), while personal resourcefulness (e.g., planning tasks) buffered the association between anticipatory grief and perceived stress (Kobiske et al. 2019). Further, age-related differences in coping were also observed. Older ADRD family caregivers described relying on faith and spirituality to manage anticipatory grief, emphasizing connection to a higher power and community, while younger caregivers reported prioritizing social connection outside the home (Duplantier and Williamson 2023). Finally, preparedness for future losses was associated with lower levels of anticipatory grief (Supiano et al. 2022), whereas certain forms of planning for future loss (e.g., funeral arrangements) were associated with higher levels of anticipatory grief (Ménard et al. 2025).

Findings regarding social support were mixed. Some studies reported that greater perceived support was associated with lower levels of anticipatory grief (Moore et al. 2020; Sánchez-Alcón et al. 2024), whereas others found no direct or moderating effects (Pérez-González et al. 2023; Malhotra et al. 2024a). Two studies found that high social support was associated with lower levels of anticipatory grief (Moore et al. 2020; Sánchez-Alcón et al. 2024). Specifically, social support was associated with lower scores in the MM-CGI-SF PS&B and W&FI subscales (Moore et al. 2020). Pérez-González et al. (2023) found that social support did not have a direct effect on anticipatory grief; however, it had an indirect effect on anticipatory grief based on the psychological health of the caregiver (e.g., symptoms of depression and anxiety, obsessive-compulsive behaviors, and hostility). Similarly, family support did not moderate the association between caregiving burden and anticipatory grief (Malhotra et al. 2024a).

### Mental health outcomes

Six studies reported that anticipatory grief was associated with greater depressive symptoms (Liew et al. 2019, 2020; Pérez-González et al. 2023; Argueta et al. 2024; Sánchez-Alcón et al. 2024; Brice et al. 2025). Depressive symptoms were associated with higher scores in the MM-CGI-SF HS&L and W&FI subscales (Sánchez-Alcón et al. 2024). Some studies found that anticipatory grief contributed to caregiver depression independently of caregiving burden. Further, it was found that anticipatory grief amplified the effect of caregiving burden on depression and predicted depression up to 2.5 years later after controlling for baseline depression (Liew et al. 2019; Pérez-González et al. 2023). These findings suggest that anticipatory grief may uniquely influence long-term depression. Further, among ADRD family caregivers with high or average anticipatory grief, loneliness was associated with higher levels of depression (Brice et al. 2025). Beyond depression, one study found that the direct effect of anticipatory grief on broader caregiver psychological health was stronger than its indirect effect through caregiving burden (Pérez-González et al. 2023).

### Interventions for managing anticipatory grief

Six studies evaluated interventions to address anticipatory grief among ADRD family caregivers. Four were randomized controlled trials (Meichsner and Wilz 2016; Meichsner et al. 2018; Risch et al. 2024; Han et al. 2025) and 2 were pre-/post-intervention studies (Paulson et al. 2024; Stevens-Neck et al. 2024). A manualized individual therapy intervention grounded in acceptance-based approaches demonstrated reductions in anticipatory grief, particularly among ADRD family caregivers actively providing in-home care (Meichsner and Wilz 2016). An online, therapist-guided writing intervention improved coping with anticipated loss and increased use of psychosocial resources (e.g., problem-solving abilities, social support, and coping) in the short term; however, these benefits were not sustained at 5-month follow-up (Meichsner et al. 2018). Acceptance and commitment therapy (ACT), delivered via telephone or videoconferencing, showed mixed findings. Telephone-based ACT improved acceptance of loss at 6-month follow-up (Risch et al. 2024), whereas a Zoom-delivered ACT intervention demonstrated within-group improvements in anticipatory grief that were maintained after 3 months, but no significant between-group differences were observed (Han et al. 2025).

Group-based and psychoeducational interventions were found to have modest benefits. A pilot grief-focused program emphasizing identity adaptation and peer support did not show significant between-group differences in anticipatory grief, however, qualitative findings suggested improved emotional processing and normalization of grief experiences (Stevens-Neck et al. 2024). Similarly, the Savvy Caregiver Program, which emphasizes dementia knowledge, preparedness, and self-care, was associated with reductions in anticipatory grief from baseline. Specifically, greater preparedness was associated with greater reductions in anticipatory grief (Paulson et al. 2024). Overall, interventions that emphasized acceptance, preparedness, and meaning-making demonstrated the most consistent improvements in anticipatory grief, although sustained effects and between-group differences were variable.

## Discussion

This scoping review synthesized evidence from 30 studies examining anticipatory grief among family caregivers of people living with ADRD and identified several consistent patterns. Caregiving-related stressors, specifically BPSD management, were associated with higher levels of anticipatory grief. Relationship changes, including loss of reciprocity and shifting roles, were key contributors to anticipatory grief. Psychosocial resources, such as coping strategies, social support, and resilience, were generally associated with lower levels of anticipatory grief; however, findings were mixed across studies. Further, anticipatory grief was consistently associated with depressive symptoms and broader psychological health, including associations with longer-term mental health outcomes independent of caregiving burden. Finally, intervention studies that assessed anticipatory grief among family caregivers of people living with ADRD were limited, but findings suggest that interventions focused on acceptance and preparedness may help mitigate anticipatory grief.

Our findings suggest that anticipatory grief can be understood as a dynamic and relationship-dependent process. This conceptualization of anticipatory grief reflects the emotional adjustments that caregivers make in response to ongoing stressors and evolving caregiving demands. Prior research demonstrates that caregivers’ day-to-day mental health is associated with daily stressors and the availability of psychosocial resources such as social support (Puga et al. 2023). Progressive losses experienced across the ADRD trajectory may contribute to changes in how caregivers perceive their role and their interactions with the person living with ADRD over time. Changes in role identity, increasing involvement in decision-making, and growing emotional distance may intensify caregivers’ negative perceptions of caregiving and adversely affect their mental health (Quinn and Toms 2018; Shrestha et al. 2022; Malhotra et al. 2024b).

Based on the Stress Process Model and the Grief-Stress Model of Caregiving, ambiguous loss, relationship changes, and role overload may be conceptualized as primary and secondary stressors within the caregiving context. Variability in exposure to these stressors over time may contribute to heterogeneity in anticipatory grief onset and severity, as caregivers experience new losses, reframe the meaning of their relationship with the person living with ADRD, or develop ambivalent feelings about their role (Cross et al. 2018). This conceptual framing highlights how ambiguous loss and caregivers’ emotional readjustment to the caregiving role creates a context in which anticipatory grief is recurrent and cumulative, with implications for long-term psychological functioning (Pearlin et al. 1990; Noyes et al. 2010).

Our review suggests that anticipatory grief may emerge from the interaction of caregiving-related stressors and caregivers’ psychosocial resources. However, broader literature has largely focused on risk factors, with relatively few examinations of how risk and resilience processes interact in the context of anticipatory grief (Barrera-Caballero et al. 2021; Gorostiaga et al. 2022; Liu et al. 2022; Magan et al. 2022; Fowler et al. 2024; Rebolo et al. 2025). Considering these processes separately may obscure dynamic mechanisms that influence caregivers’ vulnerability to anticipatory grief and mental health challenges over time.

Consistent with this perspective, spousal caregivers who co-reside with the person living with ADRD, provide care to a younger care recipient, and manage multiple BPSD may be more vulnerable to anticipatory grief. In contrast, caregivers with similar risks but who use more positive psychosocial factors, such as acceptance and adaptive coping strategies, have a closer relationship to the care recipient and are more prepared for their role, may therefore demonstrate greater resilience and lower levels of anticipatory grief. Relationship changes can also bring positive caregiving experiences, including personal growth, strengthened bonds, a sense of duty, and competence, which may further strengthen resilience and enhance perceived rewards of caregiving (Lloyd et al. 2016; Park et al. 2024). These positive caregiving experiences may help buffer the emotional impact of perceived relationship loss and may be associated with lower levels of anticipatory grief among some caregivers.

Together, these findings position anticipatory grief as emerging from the intersection of caregiving-related stress exposure and adaptive capacity. Resilience has been conceptualized as a dynamic process of adaptation shaped by the caregiving context and closely linked to caregiver mental health (Poe et al. 2023). Prior work indicates that resilience factors (e.g., adaptive coping, self-efficacy, optimism, and social support) are associated with better health outcomes, lower psychological distress, and a stronger relationship between the caregiver and the person living with ADRD (Martyr et al. 2023). Resilience factors, such as coping, have also been shown to mediate the relationship between caregiving burden and well-being (Dias et al. 2015; Clement-Carbonell et al. 2025). Within this context, resilience-related resources may shape how caregivers respond to relationship losses and caregiving stressors, potentially contributing to variability in anticipatory grief experiences.

Although social support is a well-established resilience factor, evidence regarding its associations with anticipatory grief in the present study was mixed (Dias et al. 2015; McKenna et al. 2022). The influence of social support on caregivers’ mental health may depend less on its presence and more on the type of support (e.g., emotional, informational, instrumental), adequacy, and quality (Liang et al. 2022; Zanjari et al. 2022; Acoba 2024). Prior research suggests that different forms of support may influence caregiver well-being differently. For example, instrumental support (i.e., help with a specific problem) was associated with lower daily odds of depressive symptoms, whereas emotional support was associated with higher daily odds of anxiety symptoms among ADRD caregivers (Puga et al. 2023). These findings underscore the need for more nuanced assessments of social support within anticipatory grief research.

Notably, most caregiving stress research has primarily examined caregiving burden and depression as key indicators of caregiver mental health (Koyama et al. 2017; Tian et al. 2022; Zhu et al. 2024). The findings of this review underscore the importance of considering anticipatory grief as a distinct dimension of caregiver mental health. Across the studies included in this review, anticipatory grief was associated with poorer mental health, appeared to persist across the ADRD trajectory, and was consistently linked to higher depressive symptoms. This is consistent with prior literature suggesting that grief and depression are closely related but conceptually distinct, and that depressive symptoms can obscure the unique contribution of grief-related processes to caregiver mental health (Ying et al. 2018; Sánchez-Alcón et al. 2023; Buur et al. 2024).

Although anticipatory grief shares some conceptual overlap with caregiving burden, particularly in domains reflecting emotional strain and role demands (Chan et al. 2020; Dehpour and Koffman 2023), several studies in this review reported that anticipatory grief remained associated with depressive symptoms even after accounting for caregiver burden. These findings suggest that anticipatory grief captures relationship and emotional aspects of the caregiving experience that may not be fully reflected by traditional measures of burden. Recognizing anticipatory grief as a distinct dimension of caregiver psychological distress may help inform more targeted approaches to supporting caregiver mental health.

While most interventions designed to improve caregiver mental health have primarily focused on depression and caregiving burden (Dessy et al. 2022; Sun et al. 2022; Widiyaningsih et al. 2025), relatively few have specifically addressed anticipatory grief. Some of the interventions included in this review did not identify anticipatory grief as a primary outcome, highlighting a gap in current intervention design. This gap suggests that anticipatory grief may represent an under-addressed dimension of caregiver well-being within existing intervention frameworks. To more effectively address anticipatory grief, future interventions should examine mechanisms such as acceptance, preparedness for the future, recognition, and validation of grief-related responses. Interventions that acknowledge ongoing relationship losses and normalize caregivers’ emotional responses may be particularly relevant in dementia caregiving contexts. Beyond risk-mitigating approaches, this review underscores the need for resilience-focused intervention frameworks. Incorporating psychosocial factors, such as self-efficacy, perceived gains, mastery, cultural beliefs, and family functioning, may further enhance caregivers’ adaptive capacity across the ADRD trajectory (Poe et al. 2023; Clement-Carbonell et al. 2025).

### Limitations

This review is not without limitations. First, selection bias is possible as included studies were restricted to English-language publications from selected databases. Second, consistent with scoping review methodology, study quality and risk of bias were not assessed, limiting conclusions about evidence strength. Third, small sample sizes and limited racial/ethnic diversity across studies limit the ability to capture cultural and other contextual factors influencing caregivers’ anticipatory grief. Fourth, the anticipatory grief literature is characterized by conceptual and measurement heterogeneity, including multiple definitions and assessment tools, which may limit comparability across studies. Finally, this review focused on anticipatory grief during the caregiving period and did not synthesize evidence on post-bereavement outcomes (e.g., grief after death), which is an important related area for future research.

### Implications and future directions

The findings of this review have several implications for research and practice. Healthcare providers may benefit from assessing changes in the caregiver–care recipient relationship early in the ADRD trajectory to understand how relationship dynamics contribute to anticipatory grief and caregiver well-being. Integrating anticipatory grief into caregiver assessments may help clinicians capture caregivers’ emotional experiences beyond traditional indicators of burden or depression. Future research would benefit from longitudinal designs to capture within-person fluctuations in anticipatory grief and identify contextual or psychosocial factors that exacerbate or buffer it over time. There is also a need for multicomponent interventions that explicitly target resilience-related processes associated with anticipatory grief. Finally, research with racial and ethnically diverse caregivers is essential for understanding how cultural contexts influence anticipatory grief and adaptation.

## Conclusion

This scoping review synthesized evidence on anticipatory grief among caregivers of people living with ADRD. Relationship changes across the dementia trajectory and caregiving-related stressors, particularly BPSD management, were consistently associated with higher levels of anticipatory grief, whereas psychosocial resources such as adaptive coping and social support were linked to lower levels. Anticipatory grief was also consistently associated with depressive symptoms. These findings suggest that anticipatory grief represents an important dimension of the caregiving experience that warrants greater attention in research and clinical practice. Further research is needed to better understand how cultural factors can influence the anticipatory grief experiences among racially and ethnically diverse caregivers.

## Supporting information

10.1017/S1478951526102478.sm001 Rodriguez Colmenares et al. supplementary material Rodriguez Colmenares et al. supplementary material

## Supplementary material

The supplementary material for this article can be found at https://doi.org/10.1017/S1478951526102478.
