# Supplementary material for: Anticipatory grief among caregivers of people living with dementia: A scoping review
Source: Palliat Support Care. 2026 May 6;24:e130. doi: 10.1017/S1478951526102478 (PMC13202407; doi:10.1017/S1478951526102478)
Supplement: Rodriguez Colmenares et al. supplementary material [file S1478951526102478sup001.docx]

**Supplementary Material**

Search terms and yield of articles by database

| **Database** | **Search Terms** | **Results** |
| --- | --- | --- |
| Web of Science | TS=("caregivers" OR "caregiver*" OR "care giver*" OR "carer*" OR "caretaker*") AND TS=("dementia" OR "dementia*" OR "amentia*" OR "Alzheimer*") AND TS=("anticipated grief" OR "anticipated grieving" OR "anticipatory grief" OR "anticipatory grieving" OR "anticipatory mourning" OR "pre-death grief" OR "predeath grief" OR "pre-death grieving" OR "predeath grieving") | 147 |
| Scopus | TITLE-ABS-KEY("caregivers" OR "caregiver*" OR "care giver*" OR "carer*" OR "caretaker*") AND TITLE-ABS-KEY("dementia" OR "dementia*" OR "amentia*" OR "Alzheimer*") AND TITLE-ABS-KEY("anticipated grief" OR "anticipated grieving" OR "anticipatory grief" OR "anticipatory grieving" OR "anticipatory mourning" OR "pre-death grief" OR "predeath grief" OR "pre-death grieving" OR "predeath grieving") | 101 |
| Embase | (('caregiver'/exp OR 'caregiver' OR 'care giver' OR 'carer' OR 'caretaker') OR (caregiver*:ti,ab OR 'care giver*':ti,ab OR carer*:ti,ab OR caretaker*:ti,ab)) AND (('dementia'/exp OR 'dementia' OR 'Alzheimer disease'/exp OR 'Alzheimer disease') OR (dementia*:ti,ab OR amentia*:ti,ab OR Alzheimer*:ti,ab)) AND (('grief'/exp OR 'grief' OR 'anticipatory grief' OR 'anticipated grief') OR ('anticipated grief':ti,ab OR 'anticipated grieving':ti,ab OR 'anticipatory grief':ti,ab OR 'anticipatory grieving':ti,ab OR 'anticipatory mourning':ti,ab OR 'pre-death grief':ti,ab OR 'predeath grief':ti,ab OR 'pre-death grieving':ti,ab OR 'predeath grieving':ti,ab)) | 106 |
| PubMed | ("Caregivers"[Mesh] OR caregiver*[tiab] OR “care giver*”[tiab] OR carer*[tiab] OR caretaker*[tiab]) AND ("Dementia"[Mesh] OR dementia*[tiab] OR amentia*[tiab] OR Alzheimer*[tiab]) AND (“anticipated grief”[tiab] OR “anticipated grieving”[tiab] OR “anticipatory grief”[tiab] OR “anticipatory grieving”[tiab] OR “anticipatory mourning”[tiab] OR "Pre-death grief"[tiab] OR "predeath grief"[tiab]) | 77 |
| PsycINFO | (DE "Caregivers" OR TI "caregivers" OR AB "caregivers" OR TI "caregiver*" OR AB "caregiver*" OR TI "care giver*" OR AB "care giver*" OR TI "carer*" OR AB "carer*" OR TI "caretaker*" OR AB "caretaker*") AND (DE "Dementia" OR DE "Alzheimer's Disease" OR TI "dementia" OR AB "dementia" OR TI "dementia*" OR AB "dementia*" OR TI "amentia*" OR AB "amentia*" OR TI "Alzheimer*" OR AB "Alzheimer*") AND (DE "Grief" OR TI "anticipated grief" OR AB "anticipated grief" OR TI "anticipated grieving" OR AB "anticipated grieving" OR TI "anticipatory grief" OR AB "anticipatory grief" OR TI "anticipatory grieving" OR AB "anticipatory grieving" OR TI "anticipatory mourning" OR AB "anticipatory mourning" OR TI "pre-death grief" OR AB "pre-death grief" OR TI "predeath grief" OR AB "predeath grief" OR TI "pre-death grieving" OR AB "pre-death grieving" OR TI "predeath grieving" OR AB "predeath grieving") | 22 |
| CINAHL | (MH "Caregivers" OR TI caregiver* OR AB caregiver* OR TI "care giver*" OR AB "care giver*" OR TI carer* OR AB carer* OR TI caretaker* OR AB caretaker*) AND (MH "Dementia" OR TI dementia* OR AB dementia* OR TI amentia* OR AB amentia* OR TI Alzheimer* OR AB Alzheimer*) AND (TI "anticipated grief" OR AB "anticipated grief" OR TI "anticipated grieving" OR AB "anticipated grieving" OR TI "anticipatory grief" OR AB "anticipatory grief" OR TI "anticipatory grieving" OR AB "anticipatory grieving" OR TI "anticipatory mourning" OR AB "anticipatory mourning" OR TI "pre-death grief" OR AB "pre-death grief" OR TI "predeath grief" OR AB "predeath grief" OR TI "pre-death grieving" OR AB "pre-death grieving" OR TI "predeath grieving" OR AB "predeath grieving") | 53 |

The search was conducted on May 23, 2025
